# Color Conversion of Wide-Color-Gamut Cameras Using Optimal Training Groups

**DOI:** 10.3390/s23167186

**Published:** 2023-08-15

**Authors:** Yasheng Li, Ningfang Liao, Yumei Li, Hongsong Li, Wenmin Wu

**Affiliations:** State Key Discipline Laboratory of Color Science and Engineering, School of Optoelectronics, Beijing Institute of Technology, Beijing 100081, China

**Keywords:** colorimetric characterization, color conversion, digital camera, polynomial transform

## Abstract

The colorimetric conversion of wide-color-gamut cameras plays an important role in the field of wide-color-gamut displays. However, it is rather difficult for us to establish the conversion models with desired approximation accuracy in the case of wide color gamut. In this paper, we propose using an optimal method to establish the color conversion models that change the RGB space of cameras to the XYZ space of a CIEXYZ system. The method makes use of the Pearson correlation coefficient to evaluate the linear correlation between the RGB values and the XYZ values in a training group so that a training group with optimal linear correlation can be obtained. By using the training group with optimal linear correlation, the color conversion models can be established, and the desired color conversion accuracy can be obtained in the whole color space. In the experiments, the wide-color-gamut sample groups were designed and then divided into different groups according to their hue angles and chromas in the CIE1976L*a*b* space, with the Pearson correlation coefficient being used to evaluate the linearity between RGB and XYZ space. Particularly, two kinds of color conversion models employing polynomial formulas with different terms and a BP artificial neural network (BP-ANN) were trained and tested with the same sample groups. The experimental results show that the color conversion errors (CIE1976L*a*b* color difference) of the polynomial transforms with the training groups divided by hue angles can be decreased efficiently.

## 1. Introduction

**Figure 1:**
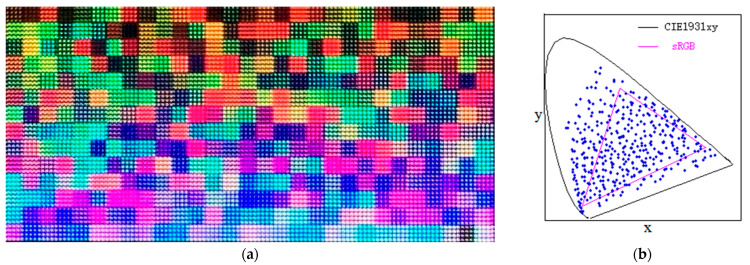
The 406 wide-color-gamut blocks displayed on a six-primary LED array display (**a**) and their (x, y) coordinates as shown by the blue dots distributed on a CIE1931xy chromaticity diagram (**b**).

The colorimetric conversion of digital cameras plays an important role not only in the field of color display but also in many other fields such as color measurement, machine vision, and so on. Today, with the rapid development of wide-color-gamut display technology, a series of color display standards with wide color gamut have been put forward, such as Adobe-RGB, DCI-P3, and Rec.2020. Meanwhile, many kinds of multi-primary display systems with wide color gamut have been demonstrated [1,2,3,4,5,6,7,8,9]. Figure 1a shows the wide-color-gamut blocks displayed on a six-primary LED array display developed by our laboratory. It can be seen from Figure 1b that the color gamut of the six-primary LED array display is much wider than that of the sRGB standard. To meet the needs of wide-color-gamut display technology, it is necessary for us to develop a colorimetric characterization method for digital cameras in the case of wide color gamut.

Until now, different kinds of colorimetric characterization methods have been developed, including polynomial transforms [10,11,12,13,14,15,16], artificial neural networks (ANN) [17,18,19,20,21,22,23], and look-up tables [24]. In 2016, Gong et al. [12] demonstrated a color calibration method between different digital cameras with ColorChecker SG cards and the polynomial transform method and achieved an average color difference of 1.12 (CIEDE2000). In 2022, Xie et al. [18] proposed a colorimetric characterization method for color imaging systems with ColorChecker SG cards based on a multi-input PSO-BP neural network and achieved average color differences of 1.53 (CIEDE2000) and 2.06 (CIE1976L*a*b*). However, the methods mentioned above were mainly used for cameras with standard color space such as an sRGB system. In 2023, Li et al. [17] employed multi-layer BP artificial neural networks (ML-BP-ANN) to realize colorimetric characterization for wide-color-gamut cameras, but the color conversion differences of some high-chroma samples were undesirable.

In addition to conventional multi-layer BP artificial neural networks, the prevailing deep learning neural networks have also been used for the color conversion of color imagers. For example, in 2022, Yeh et al. [19] proposed using a lightweight deep neural network model for the image color conversion of underwater object detection. However, the aim of the model proposed by Yeh et al. was to transform color images to corresponding grayscale images to solve the problem of underwater color absorption; therefore, it had nothing to do with colorimetric characterization. For another example, in 2023, Wang et al. [21] proposed a five-stage convolutional neural network (CNN) to model the colorimetric characterization of a color image sensor and achieved an average color difference of 0.48 (CIE1976L*a*b*) for 1300 color samples from IT8.7/4 cards. It should be noted that the color difference accuracy achieved by Wang et al.’s CNN is roughly equal to that achieved by the ML-BP-ANN of Li et al. [17] for samples from ColorChecker SG cards. However, in the architectures of the CNN proposed by Wang et al., a BP neural network (BPNN) with a single hidden layer and 2048 neurons in the hidden layer was employed to perform color conversion from the RGB space of the imager to the L*a*b* space of the CIELAB system. This indicates that the CNN proposed by Wang et al. cannot yet replace the BP neural network. Moreover, another interesting neural network model named the radial basis function neural network (RBFNN) was demonstrated for the colorimetric characterization of digital cameras by Ma et al. [19] in 2020. The architecture of the RBFNN used by Ma et al. was made up of an input layer, a hidden layer, and an output layer, and the error back-propagation (BP) algorithm was also used for the training of the RBFNN. In summary, until now, different kinds of artificial neural networks used for color conversion have been put forward; however, neural networks employing the BP algorithm continue to prevail.

In this paper, to improve the colorimetric characterization accuracy of wide-color-gamut cameras, an optimization method for training samples was proposed and studied. In the method, the training and testing samples were grouped according to hue angles and chromas, and by using the Pearson correlation coefficient as the evaluation standard, the linear correlation between the RGB and XYZ of the sample groups could be evaluated. Meanwhile, two colorimetric characterization methods—polynomial formulas with different terms and the multi-layer BP artificial neural network (ML-BP-ANN)—were used, respectively, to verify the efficiency of the proposed method.

## 2. The Problems of Wide Color Gamut

Practically, the RGB values of a digital camera can be calculated with Formula (1), where $\bar{r}(\lambda_{i})$, $\bar{g}(\lambda_{i})$, and $\bar{b}(\lambda_{i})$ denote the spectral sensitivities of the RGB channels, respectively, $\rho (\lambda_{i})$ denotes the spectral reflectance of the object, $S(\lambda_{i})$ denotes the spectrum of the light source, and $k_{r}$, $k_{g}$, and $k_{b}$ and are the normalized constants.

(1) $$\begin{array}{l} R=k_{r}\sum_{i}S(\lambda_{i})\rho (\lambda_{i})\bar{r}(\lambda_{i})\\ G=k_{g}\sum_{i}S(\lambda_{i})\rho (\lambda_{i})\bar{g}(\lambda_{i})\\ B=k_{b}\sum_{i}S(\lambda_{i})\rho (\lambda_{i})\bar{b}(\lambda_{i}) \end{array}\}$$

Meanwhile, the tristimulus of the object can be calculated with Formula (2), where $\bar{x}(\lambda_{i})$, $\bar{y}(\lambda_{i})$, and $\bar{z}(\lambda_{i})$ denote the color matching functions of a CIE1931XYZ system, and *k* is a normalized constant.

(2) $$\begin{array}{l} X=k\sum_{i}S(\lambda_{i})\rho (\lambda_{i})\bar{x}(\lambda_{i})\Delta \lambda \\ Y=k\sum_{i}S(\lambda_{i})\rho (\lambda_{i})\bar{y}(\lambda_{i})\Delta \lambda \\ Z=k\sum_{i}S(\lambda_{i})\rho (\lambda_{i})\bar{z}(\lambda_{i})\Delta \lambda \end{array}\}$$

It can be seen from Formulas (1) and (2) that there exists a nonlinear conversion between the (*R*, *G*, *B*) and the (*X*, *Y*, *Z*). In fact, different methods have been used to model the conversion from the RGB space to the XYZ space [10,11,12,13,14,15,16,17,18,19,20,21,22,23,24]. Among them, polynomial transforms and the BP artificial neural network (BP-ANN) are the most popular.

### 2.1. Polynomial Transforms

When the color sample sets ($R_{i},G_{i},B_{i}$) and ($X_{i},Y_{i},Z_{i}$) of digital cameras are given, where *i* = 1 to N, and N is the total number of samples, a polynomial transformation model can be established for converting the [*R*, *G*, *B*] space to the [*X*, *Y*, *Z*] space for the cameras.

The simplest method is a linear model, as follows:

(3) $${\left[X,Y,Z\right]}^{T}=A\;{\left[R,G,B\right]}^{T}$$

where *A* is a matrix:

(4) $$A=\left[\begin{array}{l} a_{1}\;a_{2}\;a_{3}\\ b_{1}\;b_{2}\;b_{3}\\ c_{1}\;c_{2}\;c_{3} \end{array}\right]$$

Usually, by using the color sample sets ($R_{i},G_{i},B_{i}$) and ($X_{i},Y_{i},Z_{i}$) of the cameras, and the least squares method, the elements of matrix *A* can be estimated, and then the linear transformation model Formula (3) can be established.

However, for digital cameras, the conversion between the (*R, G, B*) space and the (*X*, *Y*, *Z*) space is nonlinear, so it is necessary to adopt the nonlinear models as follows.

The square model:

(5) $${\left[X,Y,Z\right]}^{T}=A{\left[1,R,G,B,RG,RB,BG,R^{2},G^{2},B^{2}\right]}^{T}$$

The cubic model:

(6) $${\left[X,Y,Z\right]}^{T}=A{\left[1,R,G,B,RG,RB,BG,R^{2},G^{2},B^{2},R^{3},G^{3},B^{3},RGB\cdots \right]}^{T}$$

The quartic model:

(7) $${\left[X,Y,Z\right]}^{T}=A{\left[1,R,G,B,RG,RB,BG,R^{2},G^{2},B^{2},R^{3},G^{3},B^{3},RGB\cdots ,R^{4},G^{4},B^{4}\cdots \right]}^{T}$$

In summary, polynomial transforms can be expressed with Formulas (8) and (9), where *A* is a constant matrix of three lines by *n* rows, and *n* is the number of terms in a polynomial formula.

(8) $${\left[X,Y,Z\right]}^{T}=A{\left[1,R,G,B,RG,RB,BG,R^{2},G^{2},B^{2},R^{3},G^{3},B^{3},RGB,\ldots R^{4},G^{4},B^{4}\ldots \right]}^{T}$$

(9) $$A=\left[\begin{array}{l} a_{1}\;a_{2}\cdots a_{n}\\ b_{1}\;b_{2}\cdots b_{n}\\ c_{1}\;c_{2}\cdots c_{n} \end{array}\right]$$

To conclude, the elements of matrix *A* can be decided by means of the least square method, Wiener estimation method, principal component analysis, and so on. In the paper, the least squares method was used to estimate the elements of matrix *A.*

### 2.2. Artificial Neural Network ML-BP-ANN

**Figure 2:**
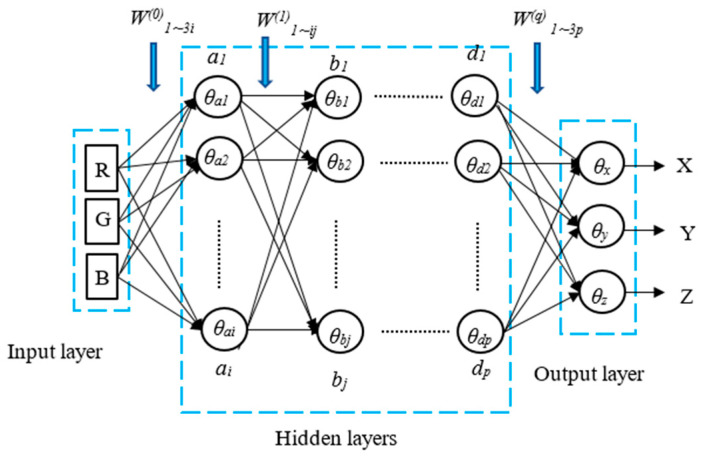
The architecture of the multi-layer BP neural network.

The multi-layer BP artificial neural network (ML-BP-ANN) can also be used to perform the conversion from the [*R*, *G*, *B*] space to the [*X*, *Y*, *Z*] space in the case of wide color gamut [17]. The architecture of an ML-BP-ANN is shown in Figure 2, where *W*^(0)^, *W*^(1)^, and *W*^(*q*)^ denote the link weights, and θ denotes the threshold of the neurons, and *a_i_*, *b_j_*, *d_p_* denote the output values of the neurons in each layer, respectively. It should be noted that the number of hidden layers and the neurons in each hidden layer can be decided based on the training and testing experiments [17], and the values of the link weights and the values of the thresholds can be decided through training. More importantly, the color sample sets ($R_{i},G_{i},B_{i}$) and ($X_{i},Y_{i},Z_{i}$) for the training should be uniformly distributed in the whole color space of the cameras.

In order to obtain desired conversion accuracy for all color samples, the training samples should be uniformly distributed in the whole color space. For instance, when the chromaticity coordinates of the color samples are distributed within a color gamut area such as the sRGB gamut triangle, the desired conversion accuracy can be easily obtained using the polynomial formulas with the terms of the cubic, the quartic, or the quantic [11,12,13,16].

However, in the case of wide color gamut, the chromaticity coordinates of the samples are distributed in the whole color space such as in a CIE1931XYZ system, and the chromas of the samples are usually bigger than those of conventional samples such as the IT8 chart, the Munsell or NCS system, the Professional Colour Communicator (PCC), X-rite ColorChecker SG, and so on. Therefore, in the case of wide color gamut, it is difficult for us to establish the color conversions with desired conversion accuracy.

To solve the problem above, we propose using an optimal method to facilitate color conversions for wide-color-gamut cameras. The method makes use of the Pearson correlation coefficient to evaluate the linear correlation between the RGB values and the XYZ values in a training group, so that a training group with optimal linear correlation can be obtained and color conversion models with desired approximation accuracy can be established.

## 3. Evaluating the Samples Using Linear Correlation

Theoretically, the Pearson correlation coefficient can be used to describe the linear correlation between two random variables. Similarly, we can use the Pearson correlation coefficient to evaluate the linear correlation between the RGB space of a digital camera and the XYZ space of a CIE1931XYZ system. Therefore, in a sample group, the ($R_{i},G_{i},B_{i}$) and the ($X_{i},Y_{i},Z_{i}$) are supposed as two random variables, respectively, the vectors ${\vec{u}}_{i}$, ${\vec{v}}_{i}$ are used to describe the coordinates ($R_{i},G_{i},B_{i}$) and ($X_{i},Y_{i},Z_{i}$), respectively, and $u_{i}$, $v_{i}$ are used to express the module of ${\vec{u}}_{i}$ and ${\vec{v}}_{i}$, respectively, as shown in Formula (10).

(10) $$\begin{array}{l} {\vec{u}}_{i}=(R_{i},G_{i},B_{i})\\ {\vec{v}}_{i}=(X_{i},Y_{i},Z_{i})\\ u_{i}=\left|{\vec{u}}_{i}\right|=\sqrt{R_{i}{}^{2}+G_{i}{}^{2}+B_{i}{}^{2}}\\ v_{i}=\left|{\vec{v}}_{i}\right|=\sqrt{X_{i}{}^{2}+Y_{i}{}^{2}+Z_{i}{}^{2}} \end{array}\}$$

Hence, the Pearson correlation coefficient (PCC) between ($R_{i},G_{i},B_{i}$) and ($X_{i},Y_{i},Z_{i}$) can be calculated using Formula (11).

(11) $$PCC=\frac{\sum_{i=1}^{n}\left(u_{i}-{\bar{u}}_{i})(v_{i}-{\bar{v}}_{i}\right)}{\sqrt{\sum_{i=1}^{n}{\left(u_{i}-{\bar{u}}_{i}\right)}^{2}}\sqrt{\sum_{i=1}^{n}{\left(v_{i}-{\bar{v}}_{i}\right)}^{2}}}$$

where *n* is the number of the samples in a sample group.

Beside the Pearson correlation coefficient mentioned above, there are many other statistical methods to analyze the relationship between ($R_{i},G_{i},B_{i}$) and ($X_{i},Y_{i},Z_{i}$). For example, the standard deviations $\sigma_{u}$ and $\sigma_{v}$ as shown in Formula (12) can be used to estimate the similarity between the vectors ${\vec{u}}_{i}$ and ${\vec{v}}_{i}$. However, the values of $\sigma_{u}$ and $\sigma_{v}$ here are variable along with the values of ($R_{i},G_{i},B_{i}$) and ($X_{i},Y_{i},Z_{i}$), so it is not a suitable method to solve our problem.

(12) $$\{\sigma_{u}=\sqrt{\frac{\sum_{i=1}^{n}{\left(u_{i}-{\bar{u}}_{i}\right)}^{2}}{n}},\sigma_{v}=\sqrt{\frac{\sum_{i=1}^{n}{\left(v_{i}-{\bar{v}}_{i}\right)}^{2}}{n}}$$

Beside Formula (12), many other methods can be used to calculate the similarity between ($R_{i},G_{i},B_{i}$) and ($X_{i},Y_{i},Z_{i}$). For example, both wavelet analysis and the gray symbiotic matrix can be used to evaluate the similarity between the chromaticity coordinate patterns of ($R_{i},G_{i},B_{i}$) and ($X_{i},Y_{i},Z_{i}$). Obviously, such methods are much more complicated than the Pearson correlation coefficient method.

## 4. Experiment

### 4.1. The Spectral Sensitivity of the Camrea

**Figure 3:**
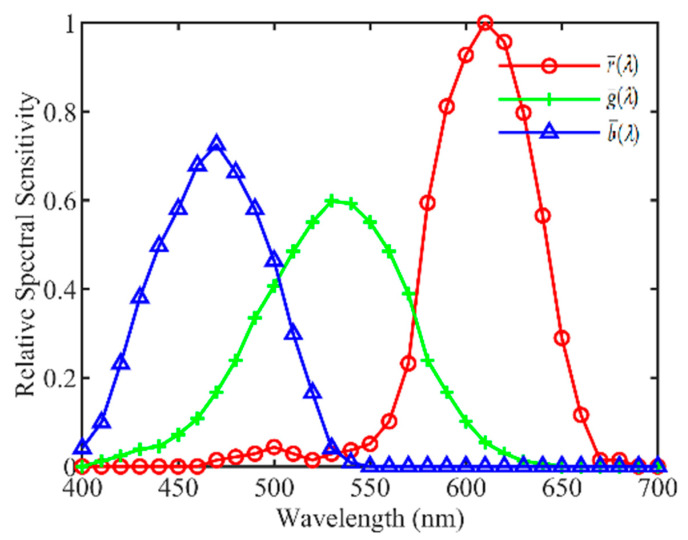
The relative spectral sensitivity curves of the RGB channels of Canon1000D.

In order to calculate the RGB values with Formula (1), it is necessary to obtain the spectral sensitivity curves of the camera. Figure 3 shows the spectral sensitivity curves of a Canon1000D camera (Canon Inc., Tokyo, Japan) from 400 nm to 700 nm at an interval of 10 nm. The experimental setups for measuring the spectral sensitivity functions $\bar{r}(\lambda )$, $\bar{g}(\lambda )$, and $\bar{b}(\lambda )$ consisted of a monochrometor (SOFN 71SW151, SOFN Instruments Co., Ltd., Beijing, China), a xenon lamp (SOFN 71LX150A, SOFN Instruments Co., Ltd., Beijing, China), an integrating sphere light equalizer, and a set of reference detectors (Thorlabs PDA100A, Thorlabs Inc., Newton, NJ, USA), wherein spectral sensitivity was characterized by the National Institute of Metrology China. Note that, in the experiment, the white point of the camera was set to D65.

### 4.2. Preparing the Sample Groups

#### 4.2.1. The Basic Samples

**Figure 4:**
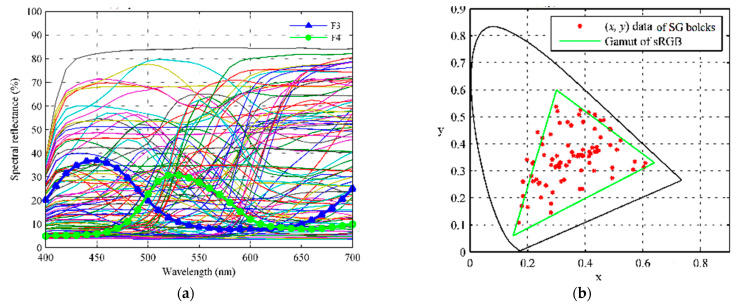
(**a**) The spectral reflectance curves of 96 ColorChecker-SG blocks measured via X-rite 7000A (de, 8°); (**b**) the (x, y) coordinates (CIE1931xy) of 96 ColorChecker-SG blocks, where the illuminant is D65.

In order to calculate the RGB values with Formula (1) and the XYZ values with Formula (2), respectively, the spectral reflectance function ρ(λ) of the samples should be prepared. In our experiment, the real color blocks of ColorChecker-SG were selected as a basic sample group. Figure 4a shows the spectral reflectance curves of the 96 ColorChecker-SG blocks measured using the spectrometer X-rite 7000A with 8° degree (X-rite Inc., Grand Rapids, MI, USA), and Figure 4b shows the CIE1931xy chromaticity coordinates of them, where the illuminant is D65. It can be seen from Figure 4b that the chromaticity coordinates of the 96 ColorChecker-SG blocks were almost distributed within the sRGB gamut triangle, therefore it is not suitable for the colorimetric characterization of the digital cameras in the case of wide color gamut.

#### 4.2.2. Extending the Samples

**Figure 5:**
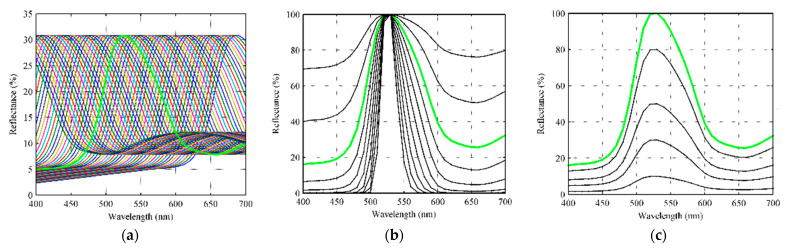

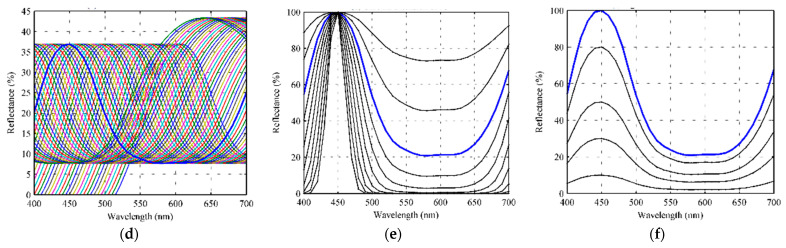
Illustrations of the spectral reflectance curves extended from ColorChecker-SG 4F and 3F, respectively: (**a**) Hue extension of F4. (**b**) Chroma extension of F4. (**c**) Lightness extension of F4. (**d**) Hue extension of F3. (**e**) Chroma extension of F3. (**f**) Lightness extension of F3.

In order to obtain samples with wide color gamut, the spectral reflectance curves of ColorChecker-SG F3 and F4, as shown in Figure 4a, were used, respectively, to generate samples distributed on the whole CIE1931XYZ space. The method for the generation of wide-color-gamut samples can be found in the reference [17]. Figure 5a,d show examples of hue extension obtained by shifting the main wavelength of the spectrum, Figure 5b,e show examples of Chroma extension obtained by sharpening or passivating the spectrum, and Figure 5c,f show examples of lightness extension obtained by scaling the peak value of the spectrum. Therefore, 5529 spectral reflectance curves were generated. By adding the 5529 curves with 96 real curves of ColorChecker-SG, we had 5625 spectral reflectance curves in total. Then, the 2813 spectral reflectance curves for the training groups were uniformly drawn from the 5625 curves, and the remanining 2812 curves were used for the testing groups.

**Figure 6:**
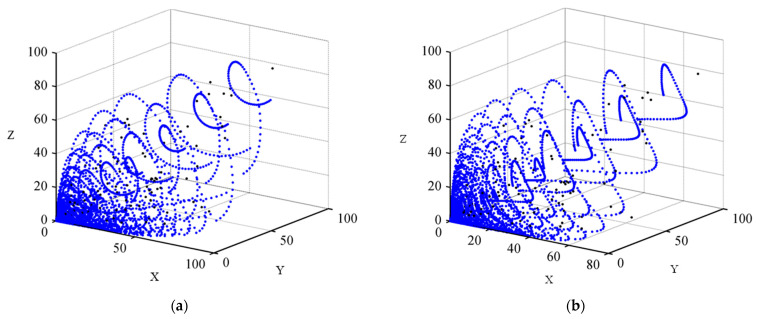
The (X, Y, Z) standard chromaticity distribution of the wide-color-gamut samples extended from F3 and F4 as shown in blue dots: (**a**) The wide-color-gamut samples extended from F3. (**b**) The wide-color-gamut samples extended from F4.

**Figure 7:**
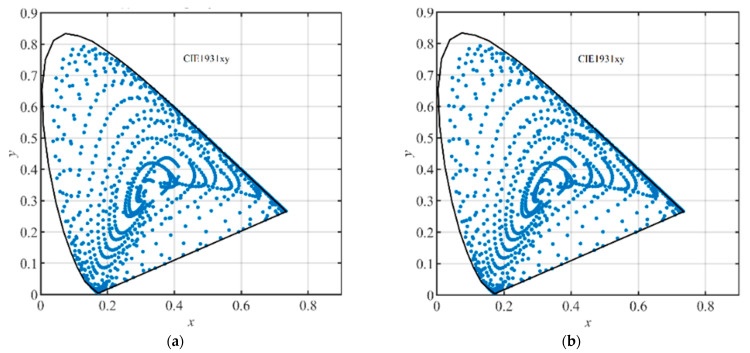
The (x, y) coordinates (CIE1931xy) of total training and testing samples as shown in blue dots: (**a**) 2813 training samples; (**b**) 2812 testing samples.

**Figure 8:**
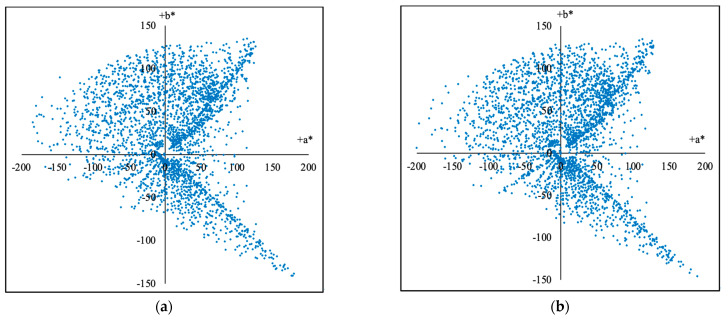
The (a*, b*) (CIELAB) coordinates of total training samples and testing samples as shown in blue dots: (**a**) 2813 training samples; (**b**) 2812 testing samples.

By using Formulas (1) and (2), the (R,G,B) and the (X,Y,Z) of the 2813 training groups and the 2812 testing groups were calculated, respectively. As results, the (X, Y, Z) distributions of the samples extended from F3 and F4 in the standard CIEXYZ color space are shown in Figure 6a,b, respectively, the (x, y) coordinates (CIE1931xy) of the 2813 training groups and the 2812 testing groups are shown in Figure 7a,b, respectively, and the (a*, b*) (CIELAB) coordinates of them are shown in Figure 8a,b, respectively. It can be seen from Figure 6 and Figure 7 that the generated samples were distributed in most of the CIE1931XYZ color space.

It should be noted that, in the experiment, the CIE illuminant D65 was used as the light source $S(\lambda_{i})$ in Formulas (1) and (2), respectively, and the R,G and B values were normalized to 255 according to the white point. However, it can be seen from Formulas (1) and (2) that the relationship between the (R,G,B) and the (X,Y,Z) was influenced by the spectrum of the light source $S(\lambda_{i})$, and if the light source $S(\lambda_{i})$ was changed, the (R,G,B) and the (X,Y,Z) of the samples should be recalculated.

#### 4.2.3. Dividing the Samples

**Figure 9:**
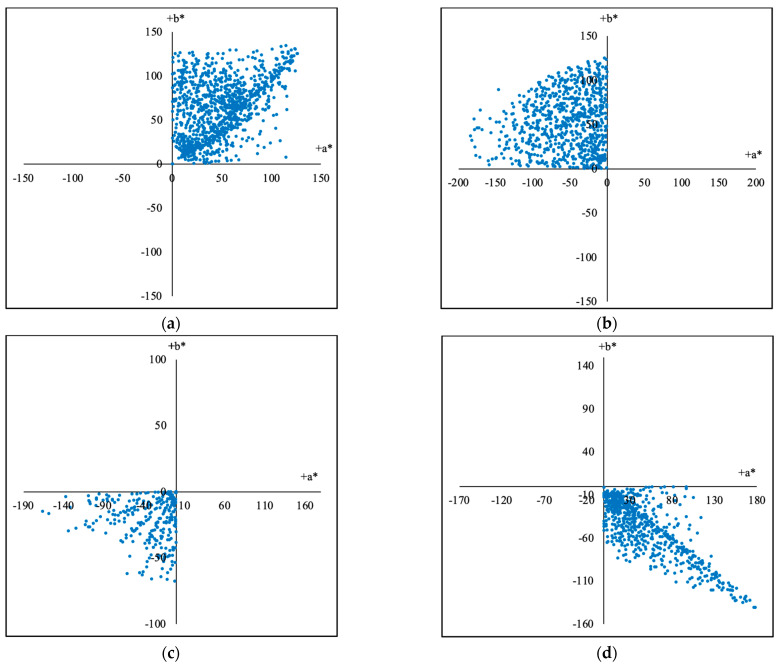
The (a*, b*) (CIELAB) coordinates of the training groups as shown in blue dots divided by the hue angles (*h*_ab_) from 2813 training samples: (**a**) The division Q1 with the hue angles from 0 to 90. (**b**) The division Q2 with the hue angles from 90 to 180. (**c**) The division Q3 with the hue angles from 180 to 270. (**d**) The division Q4 with the hue angles from 270 to 360.

In order to find the optimal training groups and testing groups, different division methods have been tried. For the first method, according to the hue angles, the samples were divided into groups of 0–90, 90–180, 180–270, and 270–360, totaling 4 parts in CIELAB color space, and the calculation formula is shown in Equation (13). As results, the 2813 training samples were divided into 4 training groups according to their hue angles ($h_{ab}$) as shown in Figure 9a–d and Table 1, and accordingly, the 2812 testing samples were divided into 4 testing groups according to their hue angles ($h_{ab}$) as described in Table 2.

(13) $$h_{ab}=\mathrm{arc}\tan(b^{*}/a^{*})$$

(14) $$C_{ab}^{*}=\sqrt{{\left(b^{*}\right)}^{2}+{\left(a^{*}\right)}^{2}}$$

**Figure 10:**
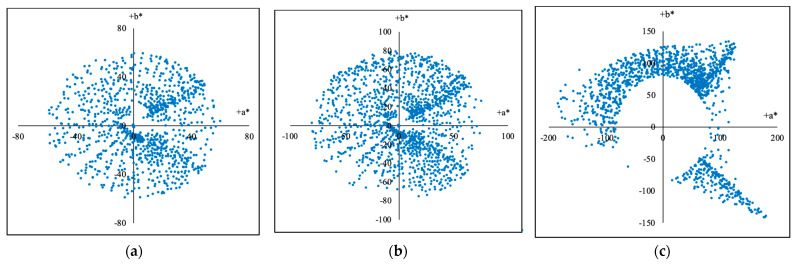
The (a*, b*) coordinates (CIELAB) of the training groups divided by the chromas (C*_ab_) from 2813 training samples: (**a**) The division C1 with the chromas from 0 to 60. (**b**) The division C2 with the chromas from 0 to 70. (**c**) The division C3 with the chromas above 70.

For the second method, according to the chromas ($C_{ab}^{*}$), the samples were divided into the groups of 0–60, 0–70, and greater than 70, totaling 3 parts, and the calculation formula is shown in Equation (14). As results, the 2813 training samples were divided into 3 training groups according to their chromas ($C_{ab}^{*}$) as shown in Figure 10a–c and Table 3, and accordingly, the 2812 testing samples were divided into 3 testing groups according to their chromas ($C_{ab}^{*}$) as described in Table 4. 

### 4.3. The Correlation Coefficients

By using Formulas (10) and (11), the Pearson correlation coefficients between the (*R*, *G*, *B*) and the (*X*, *Y*, *Z*) of different sample groups were calculated, respectively, and the calculation results are listed in Table 1, Table 2, Table 3 and Table 4, respectively. It can be seen from Table 1 that all the Pearson correlation coefficients of the training groups Q1, Q2, Q3, and Q4, which were divided by the hue angles ($h_{ab}$), are bigger than that of the 2813 training group. Similarly, it can be seen from Table 2 that all the Pearson correlation coefficients of the testing groups Q1, Q2, Q3, and Q4 are bigger than that of the 2812 testing group. It indicates that sample groups with better linear correlation can be obtained using the hue angles ($h_{ab}$). However, it can be seen from Table 3 that the Pearson correlation coefficients of the training groups C1, C2, and C3, which were divided by the chromas ($C_{ab}^{*}$), get smaller with the increase in the chromas. It indicates that their linear correlation will get worse with the increase in the chromas.

### 4.4. Training and Testing

**Figure 11:**
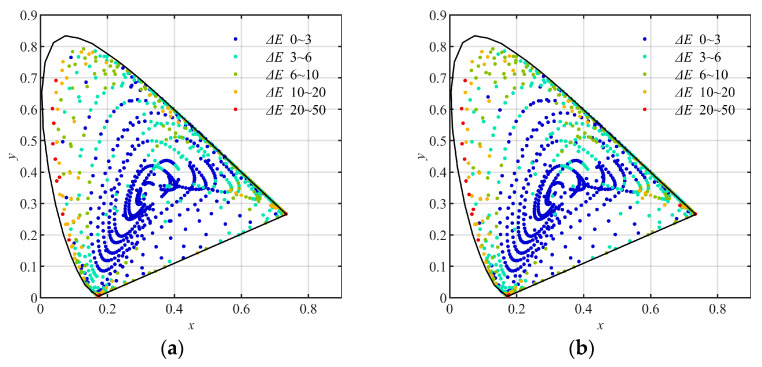
The (x, y) coordinates (CIE1931xy) of the samples converted using the quantic polynomial formula, and marked with different color difference levels: (**a**) the 2813 training samples nongrouped; (**b**) the 2812 testing samples nongrouped.

**Figure 12:**
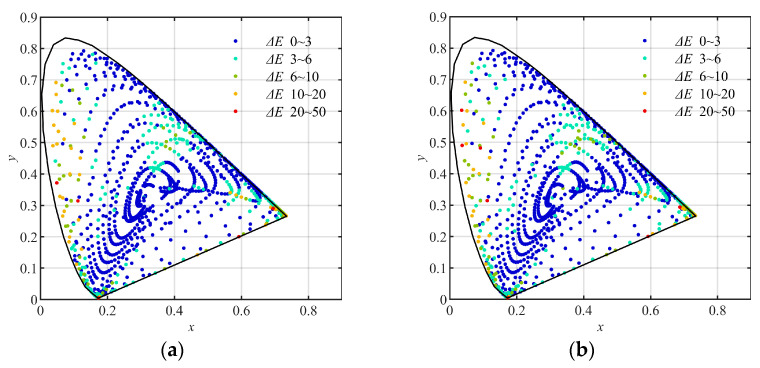
The (x, y) coordinates (CIE1931xy) of the samples converted using the quantic polynomial formula, and marked with different color difference levels: (**a**) the 2813 training samples grouped by hue angle (h*_ab_); (**b**) the 2812 testing samples grouped by hue angle (h*_ab_).

**Figure 13:**
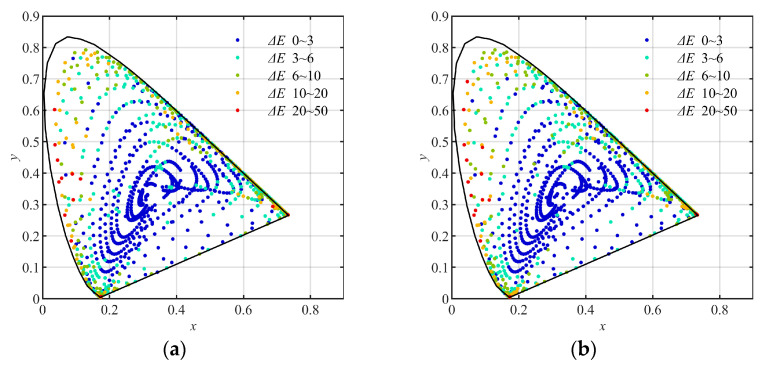
The (x, y) coordinates (CIE1931xy) of the samples converted using the quantic polynomial formula, and marked with different color difference levels: (**a**) the 2813 training samples grouped by chromas (C*_ab_). (**b**) the 2812 testing samples grouped by chromas (C*_ab_).

Using the training groups and the testing groups above, the multi-layer BP artificial neural network (ML-BP-ANN) with the architecture of 20 (neurons) × 4 (layers) and 20 (neurons) × 8 (layers), and the polynomial formulas with different numbers of terms, including the quadratic, the cubic, the quartic, and the quantic, which were described in Section 2, were trained and tested, respectively. In the training, the least square method was used to decide the elements of matrix A in Formula (9). As results, the training errors and the testing errors expressed with CIE1976 L*a*b* color difference are listed in Table 5, Table 6, Table 7 and Table 8, respectively. The distribution of the (x, y) coordinates (CIE1931xy) of the training and testing samples converted using the quantic polynomial formula, and marked with different color difference (CIE1976 L*a*b*) levels, are shown in Figure 11, Figure 12 and Figure 13, respectively.

It can be seen from Table 5, Table 6, Table 7 and Table 8 that the color approximation accuracy of the polynomial formulas depends on the number of terms in the polynomial formulas, and desirable color conversion performance was achieved using the ML-BP-ANN with the architecture of 20 × 8 and the quintic polynomial formula.

### 4.5. Discussion

(1) By comparing Table 1 with Table 5, Table 2 with Table 6, Table 3 with Table 7, and Table 4 with Table 8, respectively, we can see that for each training group or testing group, the average and the maximum color differences (CIE1976L*a*b*) are in proportion to the Pearson correlation coefficient of the samples group itself. It indicates that the color approximation accuracy of both the polynomial formulas and the ML-BP-ANN can be improved by selecting the training groups with optimal linear correlation.

(2) It can be seen from Table 1 and Table 5 that the average and maximum color differences of the group Q96 is much smaller than that of other groups because the Pearson correlation coefficient of the group Q96 is much bigger than that of other groups. It indicates that when the chromaticity coordinates of the color samples were limited within the ordinary color-gamut area, such as the sRGB gamut triangle, the desired color approximation accuracy can be easily obtained.

(3) It can be seen from Table 5 and Table 6 that the average and maximum color differences of the groups Q1, Q2, Q3, and Q4 are obviously smaller than those of the groups Q2813 or Q2812. It indicates that sample divisions by hue angles ($h_{ab}$) is an efficient method to improve color conversion accuracy in the whole color space.

(4) It can be seen from Table 5, Table 6, Table 7 and Table 8 that the average and maximum color differences of the groups C1 and C2 are much smaller than those of the groups Q2813 or Q2812, whereas the average color differences of the group C3 are bigger than those of the groups Q2813 or Q2812. It indicates that the training groups using chromas ($C_{ab}^{*}$) are not a correct choice to set up the color conversion models for the whole color space.

(5) By comparing Figure 11 with Figure 12 and Figure 13, we can see that in the low-chroma area of the CIE1931xy chromaticity diagram, the color difference levels of the samples in Figure 12a,b and Figure 13a,b are correspondingly similar to those in Figure 11a,b. This indicates that for the samples with ordinary chromas such as the group Q96, it is unnecessary to divide them into sub-groups for training.

(6) By comparing Figure 11 with Figure 12 correspondingly, we can see that, in the high-chroma area of the CIE1931xy chromaticity diagram, the color difference levels in the samples in Figure 12a,b are obviously smaller than those in Figure 11a,b. It indicates again that the sample groups using hue angles ($h_{ab}$) can be used to improve the color conversion accuracy in the whole color space.

(7) By comparing Figure 11 with Figure 13, we can see that in the high-chroma area of the CIE1931xy chromaticity diagram, the color difference levels in the samples in Figure 13a,b are correspondingly similar to those in Figure 11a,b. This indicates that the sample groups using chromas (C*_ab_) are unable to improve the color conversion accuracy in the high-chroma area of the color space.

## 5. Conclusions

In this paper, we demonstrate that the Pearson correlation coefficient (PCC) can be used for evaluating the linear correlation between the RGB space of digital cameras and the XYZ space of a CIE1931XYZ system, and can be used for selecting training groups with better linear correlation. The experimental results show that for polynomial transforms with a certain number of terms, the color approximation accuracy will be in proportion to the Pearson correlation coefficients of the training groups. The experimental results also show that using training groups divided by hue angles ($h_{ab}$) is an efficient method to improve color conversion accuracy in the whole color space. Therefore, it can be expected that better color approximation accuracy could be achieved if training samples are divided into more groups according to their hue angles.

## Figures and Tables

**Table 1:** Pearson correlation coefficients (PCC) of different training groups divided by hue angles.

Division	Q1_train_	Q2_train_	Q3_train_	Q4_train_	Q2813_train_	Q96_train_
Number of samples	1089	801	279	644	2813	96
Range of hue angle *h_ab_*	0 to 90	90 to 180	180 to 270	270 to 360	0 to 360	0 to 360
PCC	0.942	0.964	0.938	0.966	0.924	0.994

**Table 2:** Pearson correlation coefficients (PCC) of different testing groups divided by hue angles.

Division	Q1_test_	Q2_test_	Q3_test_	Q4_test_	Q2812_test_
Number of samples	1099	802	275	636	2812
Range of hue angle *h_ab_*	0 to 90	90 to 180	180 to 270	270 to 360	0 to 360
PCC	0.940	0.966	0.937	0.963	0.922

**Table 3:** Pearson correlation coefficients (PCC) of different training groups divided by chromas.

Division	C1_train_	C2_train_	C3_train_
Number of samples	1037	1488	1325
Range of chroma C^*^_ab_	0 to 60	0 to 70	above 70
PCC	0.990	0.983	0.844

**Table 4:** Pearson correlation coefficients (PCC) of different testing groups divided by chromas.

Division	C1_test_	C2_test_	C3_test_
Number of samples	1027	1491	1321
Range of chroma C^*^_ab_	0 to 60	0 to 70	above 70
PCC	0.989	0.981	0.842

**Table 5:** The CIE1976L*a*b* color differences in the training groups corresponding to Table 1.

Polynomial Formulas and BP-Ann	CIE1976L*a*b* Color Difference ΔE_ab_											
	Q1_train_		Q2_train_		Q3_train_		Q4_train_		Q2813_train_		Q96_train_	
	EV.	Max.	EV.	Max.	EV.	Max.	EV.	Max.	EV.	Max.	EV.	Max.
Quadratic	4.29	28.25	6.56	43.56	4.63	31.83	3.45	19.57	10.90	85.82	1.40	5.79
Cubic	3.52	27.93	3.09	29.79	3.92	28.56	2.86	17.87	6.50	48.76	1.08	4.76
Quartic	3.05	33.30	2.98	27.31	3.82	27.19	2.59	17.47	5.53	46.59	1.01	4.19
Quintic	2.74	29.50	2.79	27.27	3.27	25.39	1.98	21.82	4.53	79.97	0.78	3.40
Ann 20 × 4	3.04	28.76	2.87	27.35	3.25	26.12	2.01	21.16	4.66	48.77	0.82	3.52
Ann 20 × 8	2.98	27.05	2.70	26.16	3.11	24.88	1.86	20.58	4.32	45.32	0.65	3.12

**Table 6:** The CIE1976L*a*b* color differences in the testing groups corresponding to Table 2.

Polynomial Formulas and BP-Ann	CIE1976L*a*b* Color Difference ΔE_ab_									
	Q1_test_		Q2_test_		Q3_test_		Q4_test_		Q2812_test_	
	EV.	Max.	EV.	Max.	EV.	Max.	EV.	Max.	EV.	Max.
Quadratic	4.31	27.56	6.56	63.25	4.89	30.83	3.44	21.03	10.99	88.26
Cubic	3.53	28.01	3.22	43.81	4.13	26.26	2.90	17.32	6.52	55.85
Quartic	3.09	36.48	3.08	43.79	4.10	26.49	2.62	14.38	5.52	48.17
Quintic	2.80	34.26	2.92	43.27	3.74	23.59	2.10	29.68	4.57	147.08
Ann 20 × 4	2.88	34.69	3.05	43.83	3.80	23.92	2.11	29.97	4.62	47.71
Ann 20 × 8	2.72	32.91	2.83	41.65	3.62	22.19	2.02	28.68	4.15	46.61

**Table 7:** The CIE1976L*a*b* color differences in the training groups corresponding to Table 3.

Polynomial Formulas and BP-Ann	CIE1976L*a*b* Color Difference ΔE_ab_					
	C1_train_		C2_train_		C3_train_	
	EV.	Max.	EV.	Max.	EV.	Max.
Quadratic	3.23	29.30	5.75	54.51	16.43	52.78
Cubic	2.25	23.06	3.13	34.75	8.67	45.57
Quartic	2.10	21.88	2.92	29.19	7.46	44.29
Quintic	1.92	19.37	2.68	24.80	5.92	43.27
Ann 20 × 4	1.98	19.96	2.79	25.12	5.96	43.33
Ann 20 × 8	1.85	17.98	2.17	23.63	5.86	42.18

**Table 8:** The CIE1976L*a*b* color differences in the testing groups corresponding to Table 4.

Polynomial Formulas and BP-Ann	CIE1976L*a*b* Color Difference ΔE_ab_					
	C1_test_		C2_test_		C3_test_	
	EV.	Max.	EV.	Max.	EV.	Max.
Quadratic	3.21	33.12	5.83	53.37	16.69	57.22
Cubic	2.20	26.08	3.11	32.24	8.81	48.29
Quartic	2.09	43.12	2.87	27.49	7.53	46.28
Quintic	1.92	19.49	2.77	46.03	5.99	47.75
Ann 20 × 4	1.94	19.06	2.79	25.77	6.04	46.73
Ann 20 × 8	1.82	17.95	2.05	23.38	5.67	45.96

## Data Availability

Data underlying the results presented in this paper are available on reasonable request from the authors.
